# A Novel Computerized Cell Count Algorithm for Biofilm Analysis

**DOI:** 10.1371/journal.pone.0154937

**Published:** 2016-05-05

**Authors:** Mareike Klinger-Strobel, Herbert Suesse, Dagmar Fischer, Mathias W. Pletz, Oliwia Makarewicz

**Affiliations:** 1 Center for Infectious Diseases and Infection Control, Jena University Hospital, Erlanger Allee 101, 07747, Jena, Germany; 2 InfectoGnostics Forschungscampus Jena e.V., Zentrum für Angewandte Forschung Jena, Philosophenweg 7, 07743, Jena, Germany; 3 Computer Vision Group, Friedrich-Schiller University Jena, Ernst-Abbe-Platz 2, 07743, Jena, Germany; 4 Institute of Pharmacy, Friedrich-Schiller University Jena, Otto-Schott-Strasse 41, 07745, Jena, Germany; University of Kansas, UNITED STATES

## Abstract

Biofilms are the preferred sessile and matrix-embedded life form of most microorganisms on surfaces. In the medical field, biofilms are a frequent cause of treatment failure because they protect the bacteria from antibiotics and immune cells. Antibiotics are selected according to the minimal inhibitory concentration (MIC) based on the planktonic form of bacteria. Determination of the minimal biofilm eradicating concentration (MBEC), which can be up to 1,000-fold greater than the MIC, is not currently conducted as routine diagnostic testing, primarily because of the methodical hurdles of available biofilm assessing protocols that are time- and cost-consuming. Comparative analysis of biofilms is also limited as most quantitative methods such as crystal violet staining are indirect and highly imprecise. In this paper, we present a novel algorithm for assessing biofilm resistance to antibiotics that overcomes several of the limitations of alternative methods. This algorithm aims for a computer-based analysis of confocal microscope 3D images of biofilms after live/dead stains providing various biofilm parameters such as numbers of viable and dead cells and their vertical distributions within the biofilm, or biofilm thickness. The performance of this algorithm was evaluated using computer-simulated 2D and 3D images of coccal and rodent cells varying different parameters such as cell density, shading or cell size. Finally, genuine biofilms that were untreated or treated with nitroxoline or colistin were analyzed and the results were compared with quantitative microbiological standard methods. This novel algorithm allows a direct, fast and reproducible analysis of biofilms after live/dead staining. It performed well in biofilms of moderate cell densities in a 2D set-up however the 3D analysis remains still imperfect and difficult to evaluate. Nevertheless, this is a first try to develop an easy but conclusive tool that eventually might be implemented into routine diagnostics to determine the MBEC and to improve outcomes of patients with biofilm-associated infections.

## Introduction

Biofilms are the preferred sessile life form of most microorganisms exhibiting complex three-dimensional structures in which the microbes are embedded in a highly variable matrix comprising proteins, complex carbohydrates, nucleic acids and other polymers [1]. Biofilms can be formed on nearly all artificial and natural surfaces. Although biofilms play an ecologically important role in the environment, they are disturbing and harmful in urban infrastructures (e.g., tap water pipelines), industrial processes (food industry) and particularly in medicine (catheters, implants, wound infections or bronchiectasis). Matured biofilms are nearly impossible to eradicate by common chemical or drug treatments because microbes embedded in a biofilm benefit from the protective matrix environment and thus exhibit strongly reduced susceptibility to disinfectants, antibiotics and even the immune system. Because biofilms have been recognized as a growing medical problem, the research interest concerning the inhibition of biofilm formation and biofilm eradication is also increasing.

There are several methods of qualitative and quantitative biofilm analysis available, each with specific strengths and weaknesses. Quantification can be performed indirectly by staining the matrix with crystal violet (similar to Gram-staining) and subsequent colorimetric measurement of the alcoholic dye extract from the matrix. The disadvantage of this method is that the several washing steps can mechanically destroy the coherence of the biofilm matrix, resulting in loss of the biomass. Thus, this method remains imprecise and of limited use in comparative studies. More accurate quantification can be achieved by scraping the biofilm from the surface, resolving the bacteria and counting viable cells (colony forming units = CFU/mL) by serial dilution and plating on an agar medium. This is the most appropriate method to determine the effects of various treatments on biofilm-embedded microbes and to evaluate the minimal biofilm inhibitory or eradicating concentrations (MBIC or MBEC) of antibiotics or disinfectants, however this method can be hampered by so called viable but non-cultivable cells (VBNC) due to reduced metabolism in biofilms [2]. MBIC and MBEC are analogous to the minimal inhibitory concentration (MIC) of antibiotics expressing the drug resistance of planktonic cells and describe the biofilm resistance to a given treatment. Studies have shown that MBEC can be up to 1000-fold of the MIC because of the phenotypic resistance conferred by biofilm formation [3]. Because of this discrepancy, a MBIC- or MBEC- instead of a MIC-guided antibiotic treatment may help to increase clinical cure rates in biofilm-associated, difficult-to-treat infections such as prosthetic joint infections, endocarditis, osteomyelitis or even pulmonary pseudomonal infections in patients with cystic fibrosis [4]. The MBIC/MBEC assay described by Moskowitz et al. [5] quantifies the viable cells in a 96-well format but is a highly laborious and time-consuming procedure (approximately 3 days), and the results are difficult to interpret. Thus, this essay is of limited use in routine medical diagnostics.

Confocal laser scanning microscopy (CLSM) combined with fluorescent staining offers the possibility of directly investigating native biofilms in a quantitative and qualitative manner and thus has become a popular approach in biofilm studies. This method also considers the VBNC [2]. For comparative studies, the CLSM bitmap image data must be digitalized before calculating the textual and volumetric parameters, yielding comprehensive values corresponding to biomass, density or morphology. Heydorn et al. developed an advanced and freely accessible program, COMSTAT [6], for biofilm analysis that was further improved by Beyenal et al. and is known as ISA3D [7]. Both programs provide extensive but mainly qualitative analysis of the data that remains disproportionally complex and unsuitable for routine diagnostics. Moreover, the viable cells (CFU/mL) that are the best describing parameter of MBIC or MBEC are not assessed. Generally, the interpretation of such digitalized methodologies are challenging because the quantification is based on image segmentation, considering binary values for the pixels (COMSTAT) or pixel intensities (ISA3D) over a given background threshold. The primary disadvantage of binary approaches is that stained matrix compounds disturb the quantitative analysis and may lead to misinterpretations.

Therefore, we sought to develop a computer algorithm that identifies the number of viable and dead bacteria within the biofilm, and allows a fast and systematic data evaluation based on cell counts as well as few binary parameters. The presented work evaluates the performance of this novel quantitative biofilm analysis algorithm (qBA) and compares qBA to other techniques.

## Materials and Methods

### Bacterial strains and culture conditions

The bacteria strains *Pseudomonas aeruginosa* PA01, and *Escherichia coli* NBK 35218 were stored in 10% (V/V) glycerol stocks at -80°C and freshly struck on an LB-plate and grown overnight at 37°C before use. For biofilm formation, the overnight culture was diluted 1:100 in fresh Mueller Hinton (MH) medium (Roth GmbH, Karlsruhe, Germany), and 300 μL were applied in X-well Tissue Culture Chambers (Sarstedt AG, Germany) and incubated at 37°C for 24 hours without shaking to allow biofilm maturation. For crystal violet staining, the biofilms were grown in 96-well microtiter (Greiner Bio-One GmbH, Frickenhausen, Germany) plates.

The planktonic cells were carefully removed and the biofilms treated for 3.5 hours at room temperature with 200 μL PBS (phosphate buffered saline) as a control or with various concentrations of colistin or nitroxoline prepared in PBS. The antibiotic solutions were carefully removed. The biofilms were washed twice with 200 μL PBS and used for the various analysis techniques. At least three independent experiments per setting were performed.

### Crystal violet quantification of biofilms

The biofilms were stained by 0.1% crystal violet solution (125 μL per well) for 10 min at room temperature and washed twice with PBS. The microtiter plate was taped onto paper towels to remove all liquids, and the plate was air dried. Crystal violet was extracted by incubation at room temperature with 95% ethanol (200 μL of per well) for 15 min. Next, 125 μL of the ethanol extracts were transferred to fresh flat-bottomed 96-well microtiter plates, and the absorption was measured at 550 nm [8].

### Determination of viable cells as CFU/mL

Biofilms were resuspended in 200 μL PBS by scraping them from the X-Well Tissue Culture Chamber cavities. The cell suspensions were transferred into 1.5 mL reaction tubes and thoroughly mixed before they were serially diluted in PBS. Next, 100 μL of the dilutions 10^6^ to 10^9^ were plated on LB agar; the colony forming units per milliliter (CFU/mL) were determined after incubation at 37°C overnight.

### Fluorescent staining and CLSM image acquisition

The biofilms were stained using LIVE/DEAD BacLight Bacterial Viability Kit for microscopy (LifeTechnologies GmbH, Darmstadt, Germany) according to the manufacturer’s protocol.

Stained biofilms were analyzed under vital conditions using an inverse confocal laser scanning microscope LSM510 (Carl Zeiss AG) by excitation at 490 nm using the argon laser line and 40 x air objective (Carl Zeiss AG). An area of approximately 100 μm (X) x 100 μm (Y) was screened in 1 μm Z-intervals (Z-stack) at green (522 nm) and red (635 nm) channels, respectively. The pinhole was adjusted to 1 μm. The biofilm data were visualized by ZEN 9.0 software (Carl Zeiss AG). The 2D images of the green and red channel (24-bit) of each experiment were exported as two-dimensional bitmap images numbered (postfix) according to their Z-layer number with a size of 1024x1024 pixels and a resolution of 72 dpi.

### The qBA algorithm

#### General aspects and interactive input

This in-house algorithm was written in C++ programming language and is compatible with Windows (XP versions and higher) and Linux Suse. The algorithm analyzes the green (nearly all cells) and red (dead cells) images of the Z-stacks separately and evaluates the viable cells that are defined as those cells that were only stained green.

Prior image processing and analysis following image parameters must be entered: XY-resolution (thereby X = Y) and Z-resolution (distance of the Z-layers) in μm. Two training parameters, *Prolongation* and *Intensity*, must be adjusted by the user.

**Fig 1:**
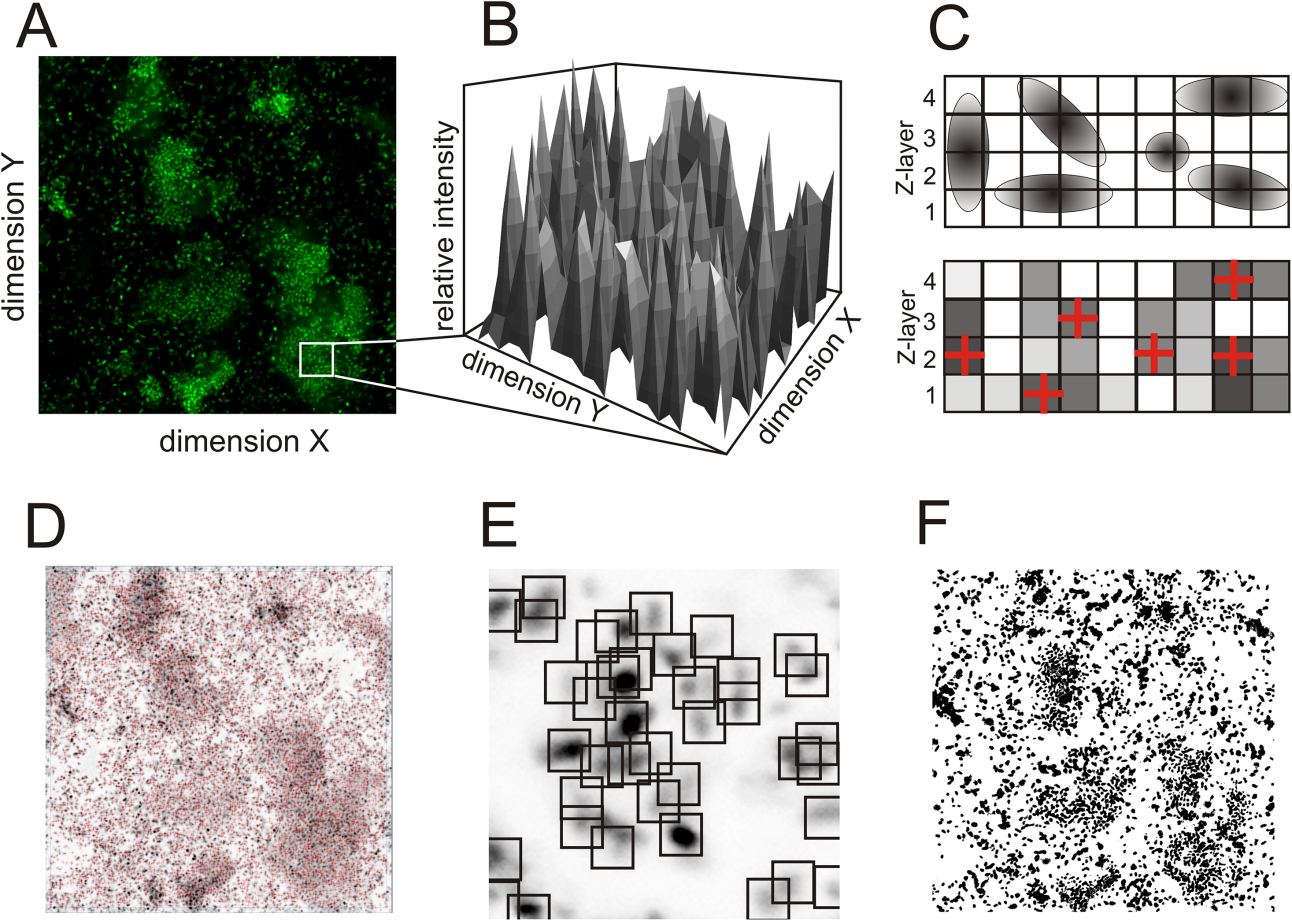
Principals of image processing and adaptive segmentation and binarization. (A) 2D CLSM image of one Z-layer; (B) Schematic example of a 3D grayscale histogram section (*g* = f[x, y]); (C) Schematic illustration of the Z-allocation of the cells; (D) 2D projection image of all Z-layers and the local grayscale maxima (indicated as red crosses); (E) Schematic illustration of the window adjustment (*w*, *w)* by prolongation and local intensity; (F) Segmented image by seeded region growing algorithm.

The qBA algorithm transforms each 2D CLSM image (Fig 1A) into an inverted grayscale 3D histogram (1 byte / pixel) scaled from 0 ≥ *g* ≥ 255 (0 = white = background, 255 = black = saturated fluorescence), in which the Z-axis represents the grayscale of each XY- pixel (Fig 1B). The gray tones describe the fluorescence intensities of the stained biofilm. The algorithm identifies local grayscale maxima upon the set parameters (described in detail below) assigning those to cell positions within one layer. Cells of neighboring layers which maxima positions are defined as overlaying (following specific conditions, as described below) are assigned to belong to the same cell and fixed to the layer with the higher grayscale value (Fig 1C). In case of identical signal intensities, the cell destination will be the lower Z-layer.

The operator must decide which Z-stack images (layers) are to be analyzed. It is recommended to start with the first bacteria containing Z-layer to avoid failures due to light reflections within the glass slide. The operator can exclude areas from the region of interest (*ROI*) (throughout entire red and green Z-stacks) by marking this in the projection image merging all Z-layers that is visualized to facilitate the entry.

After analysis various text and bitmap files are created, for example the ‘measure’-file that contains some image information and all calculations, as well as the histograms of viable and dead bacteria.

#### Adaptive basic algorithm

Contrary to other biofilm analyzing algorithms, the bacteria are counted by a novel adaptive basic algorithm. For this, the operator adjusts two parameters manually in real time: *Prolongation* (*P*) with dimension of pixels and *Intensity* (*I*) with dimension of the gray scale. Both variable parameters are pre-set (*P* = 9, *I* = 25) to allow a first allocation of the bacteria midpoints that correspond to the local maxima of the gray scale. A 2D-projection of all Z-stacks is visualized indicating local signals' maxima (*w*_*max*_) by crosses (Fig 1D). *P* depends upon the variable window size (*w*, *w*) of which *w*_*max*_ represents the highest intensity allocated in the center (Fig 1E). In case of unclear maxima and/or neighboring maxima, the connected pixels of the plateau are thinned out to achieve one pixel representing the plateau. This is computed by applying two standard morphological filters: dilation and erosion. Briefly, dilation adds pixels to the boundaries of arbitrary objects, while erosion removes pixels on objects boundaries [9]. This processing is adjusted by *I* that is defined as local contrast of the window.

#### Bacteria counting

The 2D-projection image is used to train the basic algorithm by adjusting *P* and *I* filters. The 3D-analysis is automatically performed by applying the trained basic algorithm to each image of the Z-stack.

Bacteria crossing two or more Z-layers must be offset. This is achieved by sequential evaluation of all *w*_*max*_, beginning with the uppermost image; thus, only those *w*_*max*_, are added to the bacteria count that are not connected to the neighboring layers (distance ≥ *w*/2). In this manner, the maxima of the green and red signals are assigned to XYZ-positions (centroid) and interpreted as bacteria counts, whereby the red signals directly correspond to the dead bacteria. The viable bacteria are calculated as the difference between green signals and those overlaid by red signals. The dead and viable cells counts of the image are documented in the ‘measure’-file and additionally scaled up to an area of 1 cm^2^ (*N*_*viable*_/cm^2^ and *N*_*dead*_/cm^2^).

#### Segmentation and binary parameters

The identified local maxima *w*_*max*_ are the seed points for the segmentation known as seeded region growing algorithm [10]. In this manner, the central pixel is spreading in all directions until the abort criteria considering gray tone differences in the seeded region and its neighborhood as has been described elsewhere [11] and that has been extended by maximal allowed bacteria size (diameter of 300 pixels equivalent to 30 μm). Finally, similar to COMSTAT [6], binary images (black/with) of all layers are generated (Fig 1F) visualizing the biomass. The mean thickness of the green and red stained biofilm (*D*_*green*_ or *D*_*red*_) (in μm) and its standard deviation (*SDD*_*green*_ or *SDD*_*red*_) that expresses the surface roughness are calculated from the highest (Z-position) determined pixels (black). Positions in which no biomass could be determined (white) were not considered.

The total biomass as summarized value of all layers is not provided by qBA as a quantitative parameter. The binary segmentations of the green and red images which are projected to a 2D image are used to calculate the area covered by the cells (*A*_*green*_ and *A*_*red*_) and the morphology of the biomass expressed as *Lacunarity* (*Ʌ*_*green*_ or *Ʌ*_*red*_) (not discussed in this manuscript). These parameters do not distinguish between viable and dead cells. *Lacunarity* has a character of positive values and expresses the heterogeneity of the green and red signals: a higher value indicates a higher heterogeneity (zero represents exact homogeneity). The *Lacunarity* is calculated by a box counting procedure as described previously [12].

### Simulation of biofilms

Two-dimensional images of coccal and rod-shaped bacteria cells were simulated by a random generator algorithm with varied parameters (exemplary shown in S1A and S1B Fig). For coccal cells, the short and long axes were both adjusted to 10 pixels (10 pixels = 1 μm). For rods, the short axis was adjusted to 10 pixels, and the long axis and its dimension were varied to obtain rods of different lengths and different views of the rods. The minimal distance of the centers of the cocci was defined as 5 or 2 pixels and 8 pixels for the rods. The minimal and maximal seeded gray levels (range 0–255) were set to 50 and 150, respectively. The maximal and minimal declension of the gray gradient of the cells was varied: 0, 0.25, 0.5, 0.75 and 1. Thus, 0 corresponds to homogenous filled cells (S2A Fig), and 1 corresponds to 100% of declension toward the edges of the respective gray tone with the highest tone in the center of the cell (here 50 to 150) (S2B and S2D Fig). Using non-identical values for both declension parameters resulted in cells with varying color gradients (S2C Fig). Because genuine images are rarely noise-free, we assigned a gray level of 5 as background noise. The estimated cell numbers were 10, 100, 1,000, 5,000 and 10,000. For higher counts (>1,000), the simulated cell counts per image were lower than the estimated counts because of the parameter settings and varied from 995 (instead of 1,000) to 9,495 (instead of 10,000). The images were analyzed separately and as a Z-stack simulating the green and red channels, assuming a resolution of 0.1 μm/pixel.

### Statistics

All statistics and diagrams were performed using GraphPad Prism version 6.00 for Windows (GraphPad Software, La Jolla, California USA, www.graphpad.com). Differences in parameters between the PBS and antibiotic treatments were investigated by 2way-ANOVA and a Bonferroni posttest. The correlation coefficients were determined by a Pearson test. All tests were performed applying two-sided confidence intervals of 95% and report nominal two-sided p-values.

## Results and Discussion

### Differences of qBA to other biofilm analyzing programs

Currently, one biofilm analyzing program, COMSTAT [6], is freely available. It runs as script in the commercial MATLAB environment. Similarly to qBA, it converts various ‘.tif’-files into 8 bit gray-scale bitmaps. The manual thresholding of the image stack by COMSTAT results in a three-dimensional binary (0,1) matrix, where ONE describes pixels positions with a gray tone value above or equal to the threshold, and ZERO describes pixel with values below the threshold. ISA and ISA3D [7] are further developments of COMSTAT which additionally quantify the grayscale intensity variations (0 to 255) within the images over the threshold. This approach has been suggested to improve the analysis of textural entropy of the biofilms. However, these programs are currently not available at the suggested source.

COMSTAT needs the ‘.info’-file that contains the XYZ dimensions and the pinhole size and is generated by the *Leica* microscope system or have to be individually written. In contrast, qBA algorithm do not requires additional system-specific files; the XY resolution and the Z-distance between the images are queried during the read-in. COMSTAT characterizes biofilms by ten 3D-paramaters and three additional fractal 2D-parameters; these all base on the binary matrix and express the biovolume, area occupied by the biomass and their distribution, mean and maximum biofilm thickness, and a coefficient of the roughness. This approach is therefore perfectly suitable to investigate morphological differences of biofilms.

Using qBA, no thresholding is needed because qBA identifies local grayscale maxima based on the training (*I* and *P*). These identified centroids serves as starting points for automatic binarization that, analogous to COMSTAT, is used for calculation of the biofilm thickness (*D*), roughness (*SDD*) and the fractal parameters (Ʌ). However, it has to be pointed out that qBA is primary designed for counting of the bacterial cells within a biofilm. Therefore, the most relevant parameters generated by qBA are the cell counts of viable and dead cells. Those are given as histograms and total values in the analyzed Z-stack but also extrapolated to the number of cells (*N*) per cm^2^ that is a more comfortable unit for microbiologist. The binary parameters are strongly slimmed down as they are based on the 2D projection of the Z-stack.

### Performance of qBA under simulated conditions

**Fig 2:**
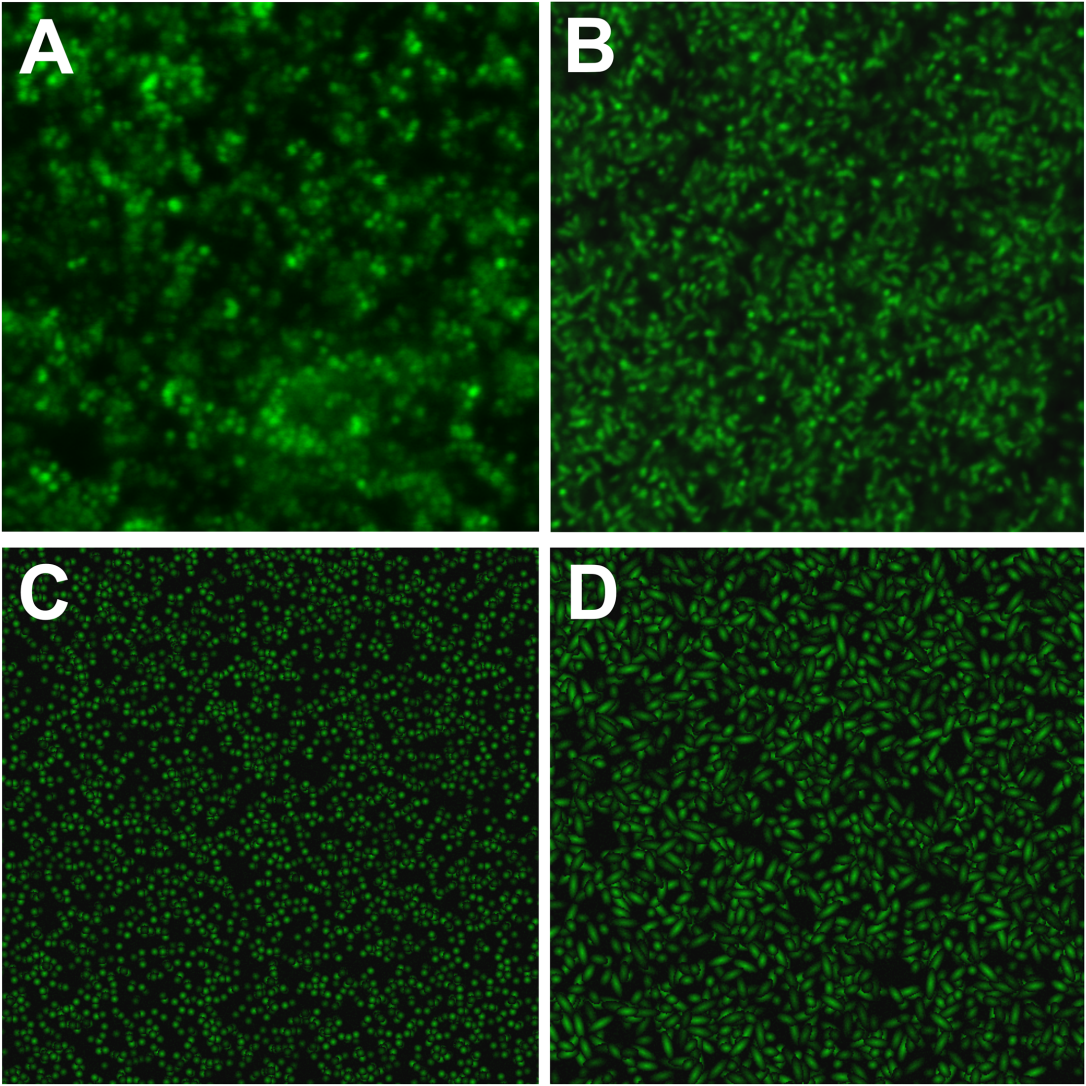
Comparison of simulated images with genuine biofilms. (A) *S*. *aureus* (cocci) biofilm and (B) *P*. *aeruginosa* (rods) biofilm. Simulations of 10,000 coccal (C) or rod (D) cells at a minimum and maximum declension = 1. All 2D-images of single layers are shown as section of similar resolution with an approximately edge length of 42 μm.

Because there exist no adequate standard to analyze the performance of qBA we first used a random generator to simulate cells (exemplary data sets are shown in S1A and S1B Fig) and evaluated the accuracy of qBA under controlled condition followed by analysis of genuine biofilms of *P*. *aeruginosa* and *E*. *coli*. Obviously, the simulated images were not fully identical to images of natural biofilms but they showed comparable densities, spread and size of the cells (Fig 2). Therefore, the simulation algorithm produced suitable 2D data set for the *in silico* analysis. However, it hast to be mentioned that the 3D resolution of the simulated Z-stacks was artificial and hardly reflected the genuine Z-distribution in biofilms as the random generator locates the cells according to mathematical patter. Despite our try to design a Z-stack composed of 2D images, the 3D performance was still difficult to evaluate *in silico*.

### Accuracy of binary and adaptive analysis depending on the cell coloration

In biofilm images, the coloration and signal intensity of the cells may vary depending on cell physiology and on the position of the cell relative to the focus of the objective (Fig 2A and 2B). Thus, we sought to analyze how these parameters influence the accuracy of qBA.

We simulated two sets of 2D images of 1,000 coccal cells with varied declension parameters. In one set, both declension parameters (minimal and maximal) were identical, and all simulated cells exhibited similar color gradients (S2A and S2B Fig). In the other set, the maximal declension was adjusted to the value of 1 to obtain images containing cells with varying color gradients (S2C Fig).

The 2D images were analyzed by qBA applying the pre-set parameters for *P* and *I*. The calculated area covered by the bacteria (*A*) and bacteria counts (*N*) were compared to the simulated values and expressed as ratios (calculated value/simulated value).

The qBA software accurately calculated the covered area for simulated bacteria with uniform graduation to 0.75 and was strongly reduced (to 70%) when the graduation reached 100% (Table 1, columns min = max). However, in genuine biofilms, the color (staining) of the cells is rarely identical in all cells. Therefore, we compared the performance in simulated images with non-uniformly graduated cell species by setting the maximal allowed graduation value to 1, varying the lowest graduation value (Table 1, columns max = 1). Here, the biomass was adequately calculated until the minimal allowed graduation reached 0.5 and was reduced to 89.2% at the minimally allowed graduation of 0.75.

**Table 1:** Accuracy of the cell counting (*N*) and the calculation of the biomass (*A*) depending on coloration of the cells.

	A		N	
Declension	max = min	max = 1	max = min	max = 1
0	0.995	1.002	1.731	1.023
0.25	0.982	0.993	0.948	0.976
0.50	1.004	0.972	0.961	0.978
0.75	1.001	0.892	0.972	0.984
1.00	0.717	/	0.979	/

The value of 1 indicates 100% accuracy.

**Fig 3:**
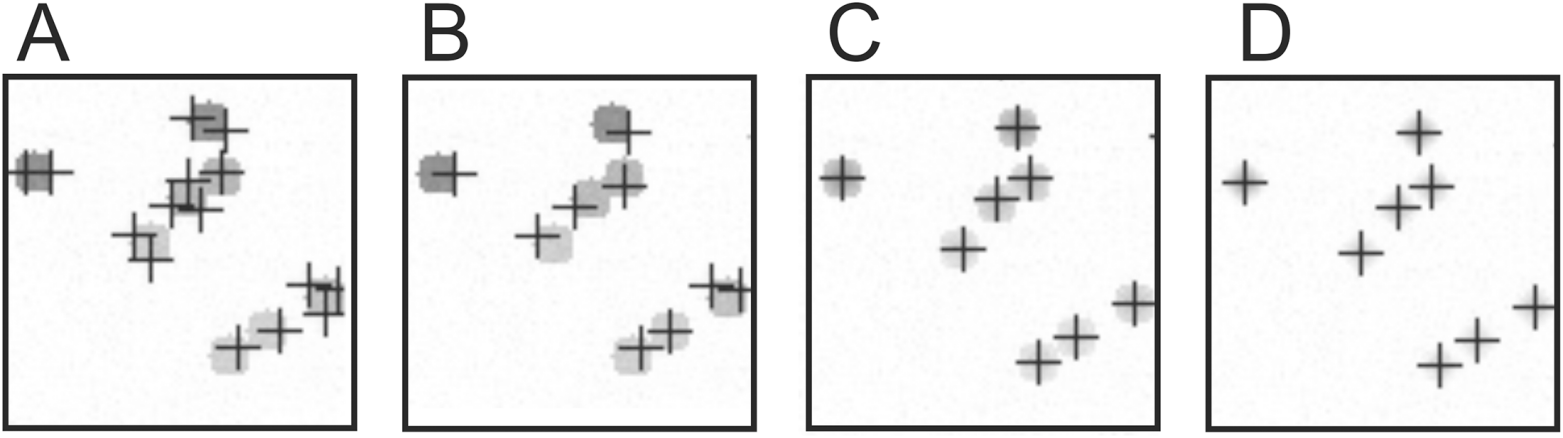
Performance of cell identification depending on various parameters. (A) No declension, *P* = 9; (B) No declension, *P* = 13; (C) Min. and max. declension = 0.5, *P* = 9, (D) Min. and max. declension = 1, *P* = 9.

The qBA software was highly accurate in counting cells with a color gradient, and the variance was within 5% for nearly all declension variants. When the cells were homogenously colored, the cell count was significantly overestimated (1.731-fold more cells identified). This, however, was a problem of the pre-adjusted *P* (9 pixels) and *I* (25) parameters. Because the cells were simulated with a diameter of 10 pixels, some of the cells were counted twice or more (Fig 3A) when there was no color declension. In this case, elevating *P* increases the accuracy to 1.31 (*P* = 11) and 1.02 (*P* = 13) (Fig 3B).

In general, the adaptive algorithm is quite accurate in allocating local grayscale maxima and thus in identifying the cells when those cells exhibit a color gradient (Fig 3C); the algorithm performs best with the highest degree of declension (Fig 3D). Because bacterial cells are rarely homogeneously colored in genuine microscopic images (Fig 2A and 2B), the algorithm appears quite appropriate for automated cell counting.

### Accuracy of bacteria counting depending in cell morphology

We analyzed the performance of the adaptive algorithm using simulated images of coccal and rod cell morphologies containing different numbers of cells (estimated 10 to 10,000 cells). The declension was adjusted to the maximum (S1 Fig). For coccal images, two sets with varying minimal distances of the cell centers were used: 2 and 5 (S1B Fig) pixels that corresponded to 80% or 50% overlapping. For the rods, the minimal distances were adjusted to 8 pixels (S1A Fig), yielding variable overlapping because of the cell shape. Two further sets of estimated 1,000 rods with varying maxima of the long axes (50 and 70 pixels) were generated. The 2D images were analyzed, varying the parameters *I* and *P* as indicated in Table 2, and the numbers (*N*) of counted and simulated bacteria were compared and expressed as ratios (counted value/simulated value).

**Table 2:** Accuracy of cell counting depending on the *Intensity* (*I*) and *Prolongation* (*P*) filters.

		*I* 25			I 17		
Cell morphology	Simulated	*P* 13	*P* 9	*P* 5	*P* 13	*P* 9	*P* 5
	*N* counts						
Cocci, distance 5*	10	1.000	1.000	1.000	4.600	4.600	4.600
	100	1.000	1.000	1.000	1.460	1.460	1.490
	965	0.926	0.979	1.000	0.959	1.016	1.042
	4,198	0.732	0.916	0.999	0.734	0.919	1.005
	7,286	0.584	0.860	0.997	0.584	0.860	0.999
Cocci, distance 2*	10	1.000	1.000	1.000	4.600	4.600	4.600
	100	1.000	1.000	1.000	1.460	1.460	1.490
	995	0.899	0.953	0.993	0.931	0.988	1.034
	4,867	0.632	0.814	0.964	0.633	0.817	0.970
	9,495	0.448	0.691	0.919	0.448	0.922	0.922
Rods, size 10x30*	10	1.000	1.000	1.000	4.500	4.500	4.500
	98	1.000	1.000	1.061	1.469	1.480	1.551
	946	0.903	0.970	1.080	0.934	1.004	1.137
	4,094	0.676	0.881	1.103	0.678	0.884	1.122
	6,963	0.539	0.801	1.075	0.540	0.802	1.088
Rods, size 10x50*	992	0.839	0.981	1.593	0.867	1.016	1.733
Rods, size 10x70*	987	0.833	1.067	2.495	0.860	1.113	2.737

A value of 1 indicates 100% accuracy, values < 1 indicate that fewer cells were counted, and values > 1 indicate that more local maxima were identified because simulated cell were present.

*The minimum distance of cocci and the size of the rods are given in pixels (0.1 μm).

When *I* and *P* were fixed at the pre-set values, images containing fewer bacteria (10 or 100) without overlap were accurately counted, independent of the cell morphology. The accuracy decreased, and cell numbers were underestimated with increasing cell density and overlap. The pre-set *P* (P 9 pixels) corresponded approximately to the diameter of the simulated coccal cells (10 pixels) and failed to differentiate individual cells within clusters. Decreasing the *Prolongation to* 5 pixels improved the accuracy even at higher densities with strongly overlapping cocci. Conversely, reducing *P* to 5 pixels led to an overestimation of rod cells with elevated rod lengths; the best counting performance was obtained at per-set *P* (9 pixels). Elevating *P to* 13 pixels greatly reduced the number of identified cells of images with high cell density.

Reducing *I* to 17 at *P* of 9 did not improve the accuracy of counting images with higher cell density (>1,000 counts) compared to the pre-set parameters but strongly distorted the results of images with lower cell density. For the image containing only 10 cells, 4.6-fold more cocci and 4.5-fold more rods were estimated, indicating that in porous biofilms, local background noise (here gray level 5) may be interpreted as cells when the *I* filter is set to low. By reducing *P* to 5 pixels, the accuracy could only be improved for images with high cell densities (>4,000 cells).

The simulation experiments indicated that the most critical parameter for proper identification of the bacterial cells is given by the *Prolongation*. The reduction of *Intensity* may slightly improve the results if high densities of bacteria are present. However, this may be a random effect because of local maxima of the background noise. Therefore, a critical real-time adjustment should be performed using the qBA software.

### Accuracy of cell counts in a 3D setting

We simulated two Z-stack sets using 2D images of coccal cells with a diameter of 10 pixels. Thus, in one set (Z-stack A), the minimal cell distance was adjusted to 5 pixels, and for the other set (Z-stack B), to 2 pixels. The cell numbers increased with the increasing Z-layer. Because the random 2D generator spreads the bacteria at fixed positions, the estimated total bacteria number (*N*) of the Z-stacks was given by the Z-layer with the highest bacteria density (uppermost Z-layer). Thus, for Z-stack A, the *N* was 6,264; and for Z-stack B, the *N* was 9,495. The estimated cell number per Z-layer was determined by the cells’ differences between the simulated 2D images. The obtained histograms (counts per Z-layer) and the total bacteria counts were compared with the estimated values and expressed as ratios (Table 3).

**Table 3:** Accuracy of cell counting per Z-layer and of the total cell number of simulated 3D biofilms depending on *I* and *P* filters.

			*I* 25		*I* 17	
Z-stack	Z-layer	estimated *N*	*P* 9	P 5	*P* 9	*P* 5
A	0	10	1.000	1.000	4.600	4.600
	1	90	1.000	1.000	1.489	1.544
	2	845	0.985	1.020	1.015	1.064
	3	2,901	0.862	1.095	0.859	1.100
	4	2,418	0.485	1.209	0.483	1.211
	total	6,264	0.735	1.127	0.750	1.150
B	0	10	1.000	1.000	4.600	4.600
	1	90	1.000	1.000	1.489	1.544
	2	895	0.931	0.987	0.960	1.028
	3	3,872	0.663	0.897	0.659	0.902
	4	4,628	0.255	0.680	0.253	0.681
	total	9,495	0.493	0.801	0.502	0.816

Compared with 2D imaging, in the 3D setting, the cell numbers were underestimated for Z-layers with higher cell densities (> approximately 3,500 cells) and increasing degree of overlapping at the pre-set *I* and *P* filters but could be partially restored by reducing *P* to reach a cell density of approximately 4,500 cells/Z-layer. Higher densities led to impaired 3D analysis with strongly overestimated (120%) or underestimated (68%) cell counts. Because we rarely observed densities of more than 5,000 cells per image (102 μm x102 μm) in natural 24 h-grown biofilms the inaccuracy at higher densities remains acceptable for most diagnostic issues.

In addition, we used an image with 100 cocal cells (distance 2) to generate images that were moved by 5 and 10 pixels and count sets of two or three of these images by qBA to analyze how the fixed *w*/2 distance performs in allocating cells spanning over two layers. As expected, the counted cell numbers depended on *P* that corresponds to *w*/2. Cells of two layers shifted by 10 pixels (S3A Fig) were recognized as separate cells at *P* ≤ 9. A distance of 5 pixels was interpreted as similar cells and the cells were estimated to the lower layer at *P* ≥ 5 (S3B Fig). Analyzing three images with cells moved by 5 pixels between every layer resulted in an estimated 100 cells in the lowest layer and 100 cells in third layer at *P* ≤ 9 (S3C Fig).

This was performed on simulated images where the grayscale maxima of the shifted cells were identical. It remains difficult to predict how the Z-allocation performs in real biofilms because the signal intensities of cells spanning over two or three biofilm layers usually differ. Moreover, using real biofilm images screened in 1 μm Z-distances an adjusted *P* value of >1 would result in a w/2 of >0.5 μm and therefore would lead to underestimation of the Z-overlapping cells that would be assigned to one cluster. The w/2 parameter should be designed as a variable, independent of *P* but related to the Z-distance.

#### Accuracy of viable cell counts

The LIVE/DEAD BacLight Bacterial Viability staining bases on two different fluorescent dyes, SYTO9 and propidium iodide, are both specific for nucleic acids; however, only SYTO9 is membrane-permeable. The green SYTO9 dye stains all bacteria cells, including dead cells that additionally incorporate the red propidium iodide dye because of lost membrane integrity. Thus, in confocal laser scanning microscopy (CLSM), the majority of the dead cells yield signals in both red and green channels. To eliminate the double counting, an algorithm was incorporated into qBA to localize cells that exhibit local maxima of both channels and to eliminate those cells from the viable count.

We simulated this case including one red channel image containing 995 bacteria (Z-layer 2) into a Z-stack of coccal cells (10 pixel diameter, declension of 1 and minimal cell distance of 5 pixels) and estimated the numbers of viable cells per Z-layer and the Z-stack (Table 4). Thus, Z-layer 0 contained 10 simulated green cells and no red cells that correspond to 10 viable and 0 dead cells. Z-layer 1 contained 100 simulated green and no red cells, corresponding to 0 estimated viable and 0 dead cells. Z-layer 2 contained 995 simulated green and 995 red cells, corresponding to 0 viable and 995 dead cells. Z-layer 3 contained 4,867 simulated green cells and no red cells, corresponding to 3,772 estimated viable and no dead cells. Z-layer 4 contained 9,495 simulated green and no red cells, corresponding to 4,728 estimated viable cells and 0 dead cells.

**Table 4:** Accuracy of cell counts of a simulated 3D biofilm containing 995 dead cells.

	estimated N		*I* 25		*I* 17	
Z-layer	viable	dead	viable	dead	viable	dead
0	10	0	1	(0)*	4.6	(1)*
1	0	0	(0)	(0)*	(49)	(0)*
2	0	995	(0)	0.993	(0)*	1.034
3	3,772	0	0.920	(0)*	0.926	(1)*
4	4,728	0	0.660	(0)*	0.667	(0)*
total	8,510	995	0.779	0.993	0.792	1.036

* Certain estimated values were zero; therefore, corresponding ratios could not be calculated and the identified cell counts are instead indicated in parentheses.

The simulated 3D biofilm was analyzed at *P* = 5 pixels, varying the intensity (*I* 25 or *I* 17) and the results of the histograms; the total counts of viable and dead bacteria were compared to the estimated values; and the accuracy was expressed as a ratio (counted value/estimated value) (Table 4).

For both *I*-filter adjustments, the accuracy of the viable cell count was approximately equal compared with the analysis of the corresponding Z-stack B (Table 3) containing only the green channel. Similar to the previous analyzes, images containing fewer cells were overestimated at a lower *I* value (of 17) because of the background noise. Summarizing the performance, the qBA software adequately identified the dead bacteria and subtracted those bacteria from the green data set to obtain the viable cell number.

### Performance of qBA using genuine biofilms

#### Accuracy of the adaptive analysis compared to manual counting

*P*. *aeruginosa* and *E*. *coli* biofilms were grown for 24 hours, washed with PBS and scanned by CLSM. Z-layers of the glass slide and below were ignored to avoid misinterpretations because of reflecting fluorescence.

**Fig 4:**
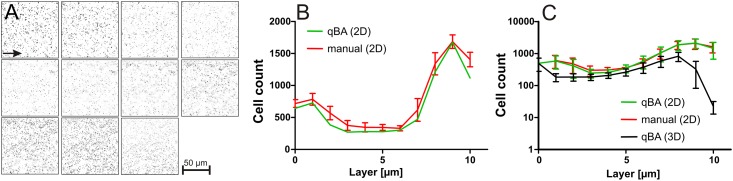
Comparison of manual counting and automated counting by qBA. T. (A) Processed images of a *P*. *aeruginosa* biofilm Z-stack. (B) Three manually and individually determined bacteria numbers (red line) per layer compared to individual counting by qBA (2D, green line). (B) Three different biofilms of *P*. *aeruginosa* were analyzed manually (red line) and by qBA (2D, green line and 3D, black line). In general the cell distribution within those three independent grown biofilms exhibited a minor deviation.

**Fig 5:**
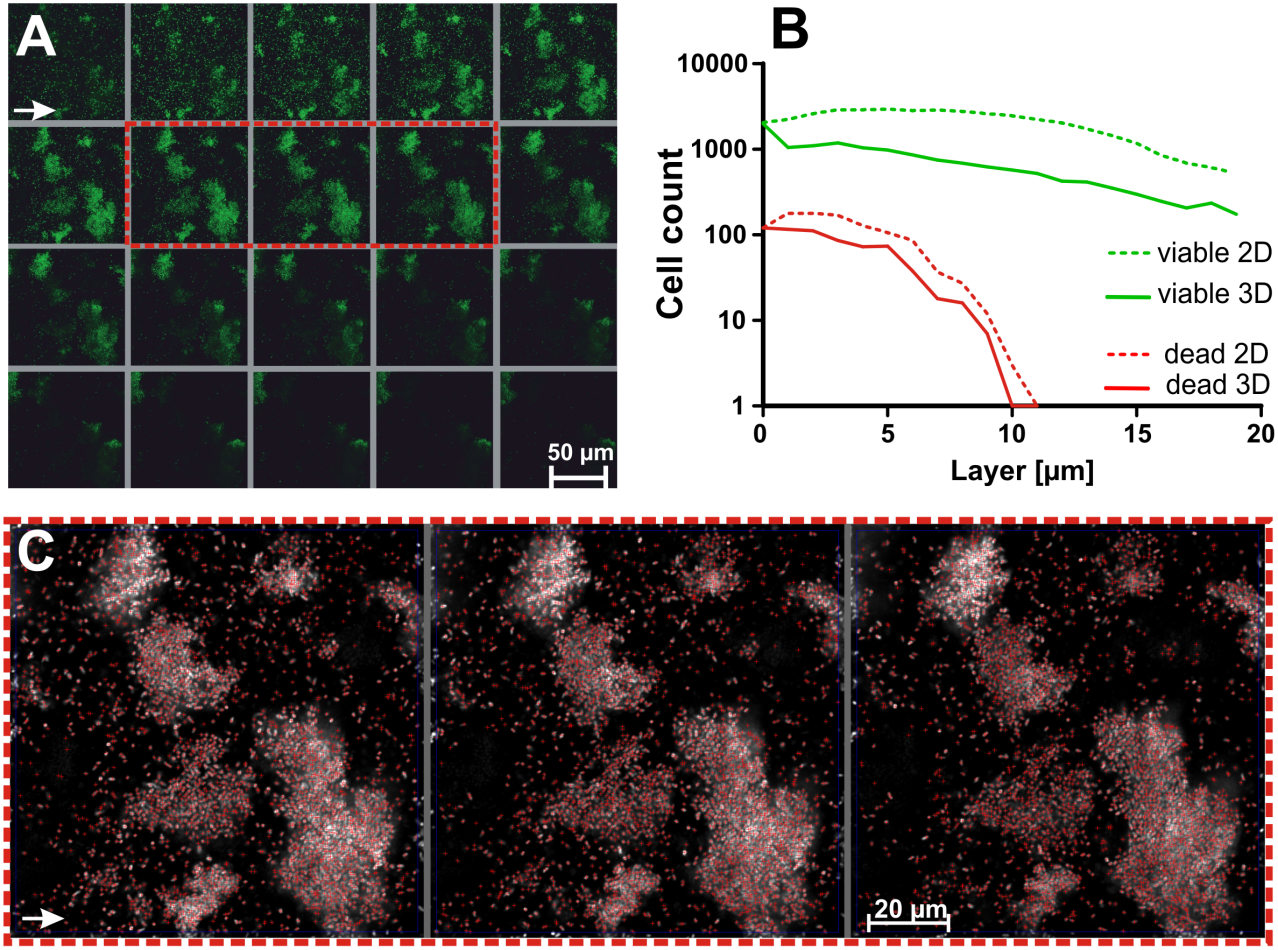
Comparison of a 2D and 3D analysis by qBA of an *E*. *coli* biofilm. (A) Analyzed biofilm layers scanned by CLSM (green and red channels overlapping). (B) Histogram of the viable (green) and dead (red) cells estimated in a 2D (dotted lines) and 3D (solid lines) setting. (C) Allocated (red crosses) local grayscale maxima in three neighboring layers (as indicated by the red dotted square in A). Biofilm images in A and B were processed by increasing the intensity and contrast of the signals for illustrative purpose.

The biofilms of *P*. *aeruginosa* exhibited a lower density and the cells were more evenly distributed comparing to *E*. *coli* that tended to form dense clusters (Figs 4 and 5). The green Z-stack images of the *P*. *aeruginosa* biofilms (n = 3) were processed to increase the visibility of the signals for the human eye that is not adapted to a 255 tone scale: change to grayscale, colour inversion, and increase of the colour depth and contrast (Fig 4A). It remained impossible to manually assign the red stained cells to the corresponding green stained cells; therefore the red images were ignored. All Z-stacks were counted manually and by qBA (in 2D and 3D setting *I* = 17 and *P* = 9). One of the Z-stacks was manually counted by three different persons (Fig 4B, red line) indicating non-significant (P > 0.05) and relatively low individual deviation (Fig 4A, non-logarithmic Y-axis to visualize the low differences). The comparison of the manual counting with the cell numbers per Z-layer determined by qBA (2D analysis) revealed a high accuracy of 1.02 ± 0.13 in average (with a 95% confidence interval of 0.98 to 1.07) between qBA and the manual analysis.

The results of the 2D analysis (manual and by qBA) of three *P*. *aeruginosa* biofilms were compared with the qBA-histograms obtained in a 3D setting (Fig 4B). Compared to the cell count of individual Z-layers (Fig 4C, green lines), the cell numbers per Z-stack were lower when counted by qBA in a 3D setting (Fig 4, black lines), because qBA assigns the cells to the layer were they exhibit the highest signal intensity. Similarly to human eye, the algorithm identifies local grayscale maxima thus fading signals in neighboring biofilm layers likely leading to overestimations in a 2D setting. However, despite the reproducibility, it is difficult to estimate if the cells were properly recognized and assigned in the 3D setting because it remains impossible to identify the Z-position of the individual cells by eye and thus a manual 3D comparison of the biofilms was technically unfeasible. Despite this drawback, the transition zone between the biofilm and the planktonic bacteria as observed by eye was clear visible in the 3D setting as a rapid downward curve in the histograms allowing a clear definition of the maximal thickness of these natural biofilms being approximately 9 μm. We are aware that the 3D analysis is still imperfect and have to be further improved, but for these biofilms exhibiting a low cell density our subjective impression was that the CLSM images were acceptably reflected by the 3D histograms.

The accuracy of the Z-allocation of bacteria cells in inhomogeneous and clumping biofilms was investigate in an *E*. *coli* biofilm (Fig 5A). The estimated cell numbers of the 2D and 3D setting differed stronger (Fig 5B) indicating that a proper Z-allocation of the bacteria remains more difficult to resolve by the set w/2 in a biofilm with cell clusters, despite the local grayscale maxima of each Z-layer were quite accurately allocated (Fig 5C). The viable cells in the 3D-histogram were most likely underestimated, and those of the 2D-histogram overestimated. It remains unfeasible to precisely identify the actual number of the cells manually thus the failure could not be calculated.

#### Application of qBA to analyze the effects of antibiotic treatment

To investigate if the current algorithm might be suitable for diagnostic issues, we treated genuine biofilms with two antibiotics and compared the obtained data of the untreated and treated biofilms as well as to other methods.

**Fig 6:**
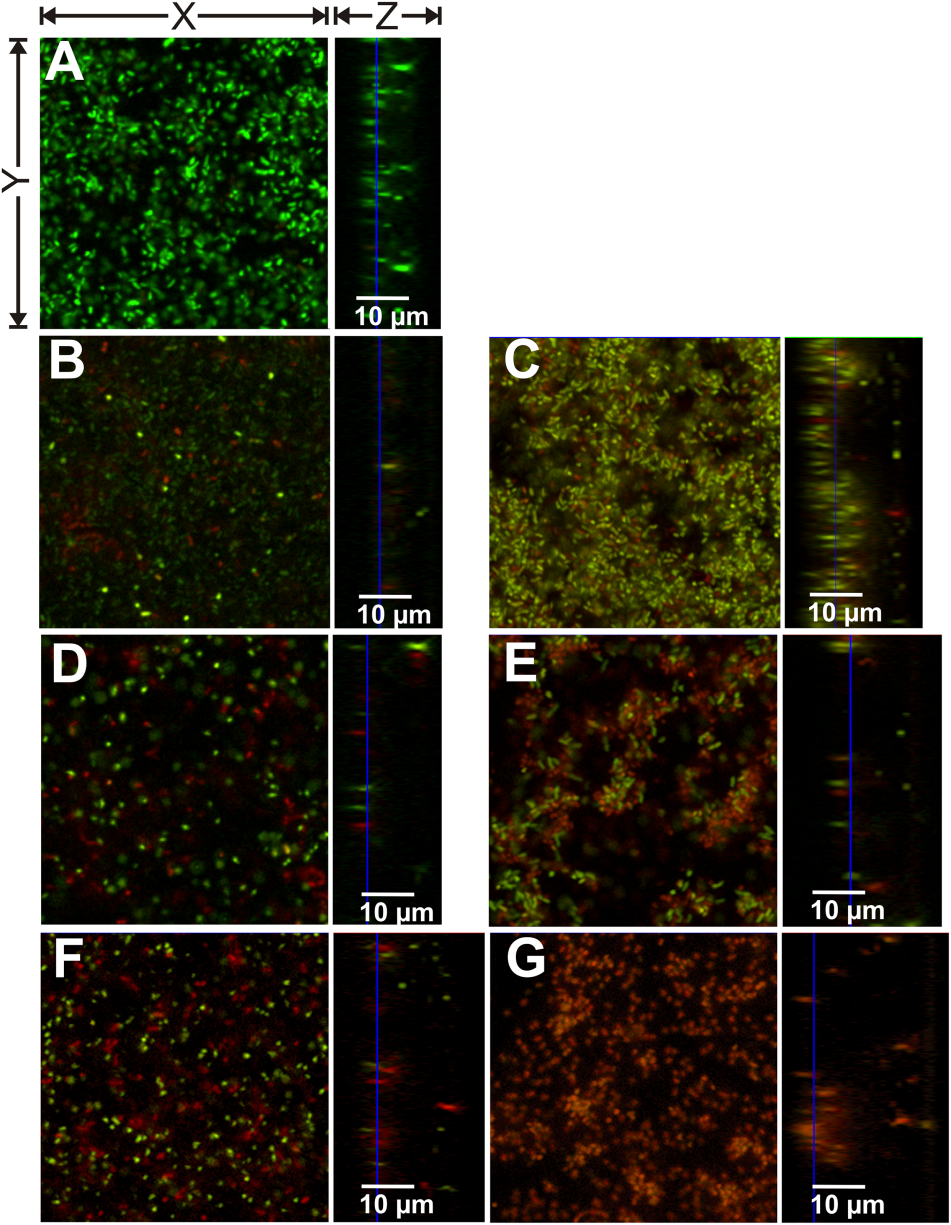
CLSM images of *P*. *aeruginosa* PA01 biofilms after 24 hours growth treated with PBS or nitroxoline or colistin for 3.5 hours. (A) PBS treatment; (B) 160 μg/mL nitroxoline; (C) 160 μg/mL colistin; (D) 320 μg/mL nitroxoline; (E) 320 μg/mL colistin (F) 640 μg/mL nitroxoline; (G) 640 μg/mL colistin. Viable cells are visible in green (SYTO 9) and dead cells in red (propidium iodide). All images present only sections of approximately 50 x 50 μm (X x Y) and variable Z-sizes (depending on biofilm thickness).

**Fig 7:**
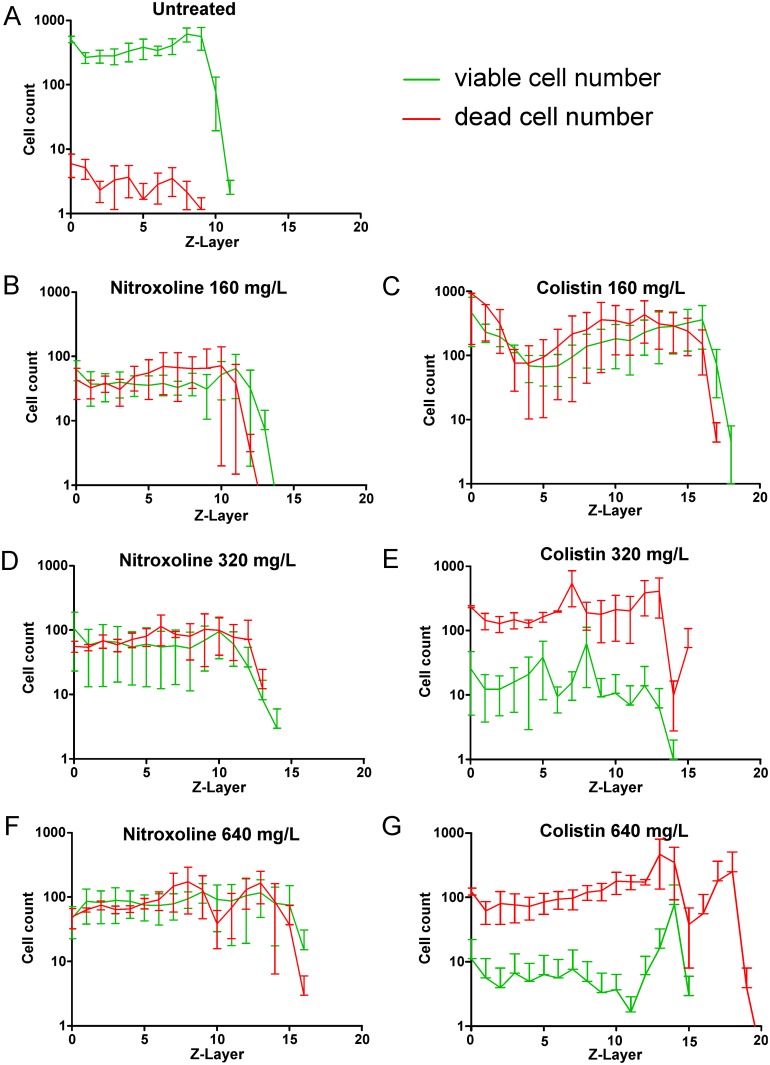
Histograms of viable and dead cells of *P*. *aeruginosa* biofilms treated by PBS or various concentrations of antibiotics. (A) PBS treatment; (B, D, F, H) Nitroxoline treatment and (C, E, G, I) colistin treatment (corresponding to Fig 6). Viable cells are represented by green lines and dead cells in red lines. Error bars indicate the standard error of the mean (SEM) for three independent experiments. Concentrations of antibiotics are indicated above the corresponding histograms.

We have previously shown that nitroxoline and colistin exhibit visible biofilm-eradicating activities [13]. Therefore, we chose these to two antibiotics to treat *P*. *aeruginosa* biofilms and to evaluate if specific effects can be observed by qBA. The biofilms were grown for 24 hours and subsequently treated with PBS or antibiotics (colistin or nitroxoline) for 3.5 hours and scanned by CLSM (Fig 6). The qBA analysis was performed adjusting *I* to 17 and *P* to 9. The distribution among the layers of the viable and dead bacteria was depicted by histograms (Fig 7).

In the PBS-treated biofilms, the number of viable bacteria was approximately 2-log magnitudes higher than the dead bacteria (Fig 7A). The overall number of bacteria and the proportion of viable cells decreased within the deeper biofilm layers as expected. It is known that in deeper layers, the microenvironment becomes unfavorable because of decreased nutrients and oxygen concentrations; thus, the numbers of persisters and dead cells increase [1, 14].

**Fig 8:**
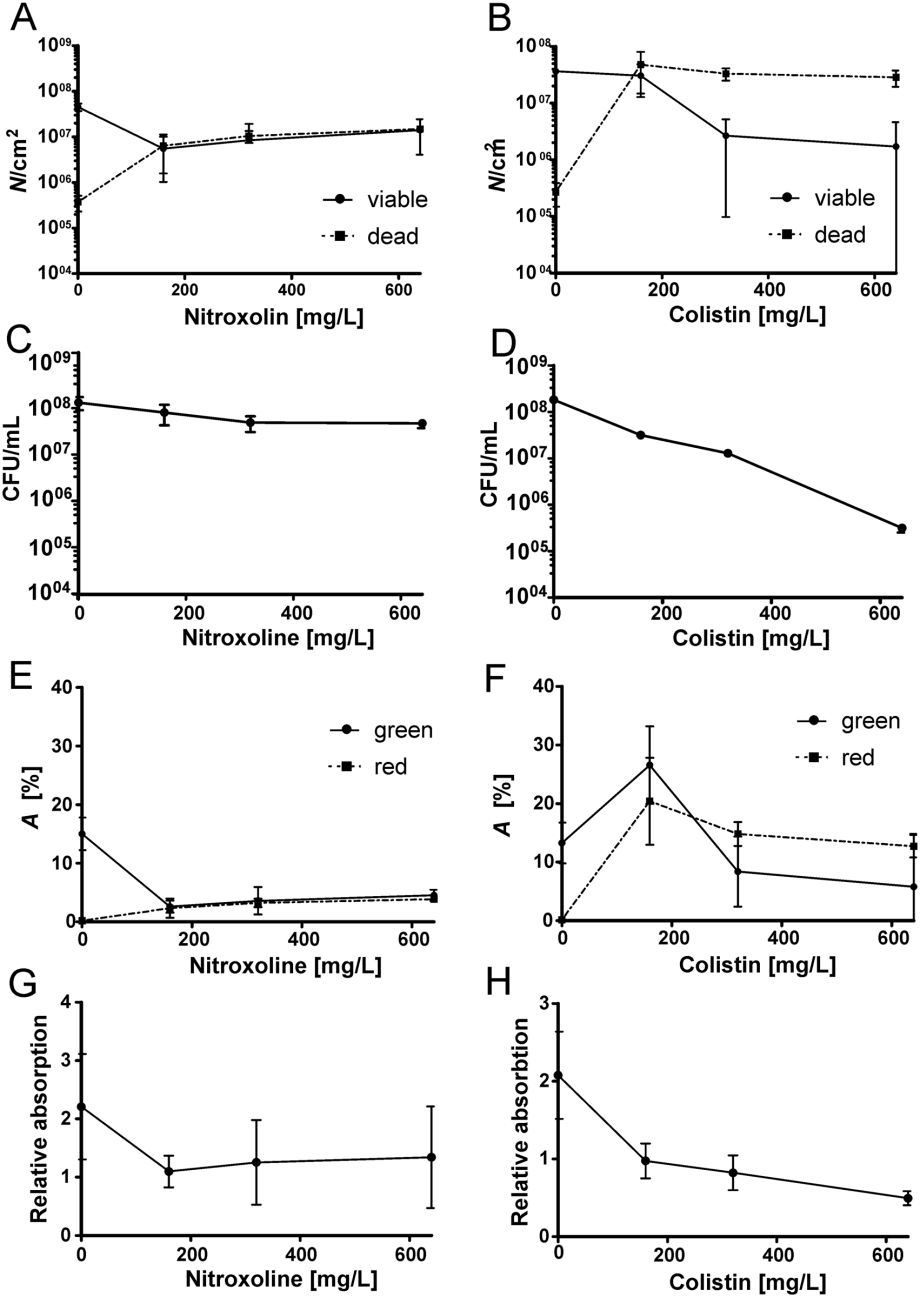
Concentration-response activities of nitroxoline and colistin against *P*. *aeruginosa* biofilms measured by different methods. (A-B) Direct count (*N* /cm^2^) of viable and dead bacteria by qBA; (C-D) Determination of viable cells on agar (CFU/mL); (E-F) Direct determination of the area (*A*) covered by green- and red-stained bacteria by qBA; (G-H) Crystal violet absorption.

The effect of nitroxoline was similar for all tested concentrations. Nitroxoline treatment resulted in a reduction of viable cells and a simultaneous increase in dead cells by one log magnitude (Fig 8A). This confirmed a primarily bacteriostatic effect of nitroxoline [15] with weak bactericidal mode of action as recently observed for *Haemophilus influenzae* [16].

In a previous work, we used 200 I.U./mL colistin (10 mg/L) for biofilm treatment, and the effect on the biofilm was rather weak. Colistin is an amphiphilic polymyxin and is known to interact with lipoid compounds and to induce instability and pore-formation in bacterial membranes [17]. In the present work, we used higher concentrations of colistin (160 mg/L to 640 mg/L), and the bactericidal effect was substantially greater and increased in a concentration-dependent manner (Fig 8B). A reduction of viable cells by approximately 1.5-log magnitudes and an increase of dead bacteria by 2-log magnitudes were achieved at 640 mg/L colistin. In line with these results, the MBEC, defined as a concentration that is required to completely (≥99.9%, or 3-log magnitudes) eradicate viable cells, of colistin for *P*. *aeruginosa* biofilms was determined to be 1024 mg/L [18].

Notably, the maximum thickness (Fig 7B to 7G) and the mean thickness (*D* ± *SDD*) (S4 Fig) of the antibiotic-treated biofilms, including both viable and dead cells, increased compared with the PBS-treated biofilms indicating possible impact of nitroxoline and colistin on the biofilm matrix. Nitroxoline is a chelating agent of divalent cations and is supposed to interact with magnesium that stabilizes the polysaccharides of the outer membrane and the biofilm matrix [19]. Chelating agents such as EDTA or heparin have been shown to be clinically effective as anti-infective lock solutions to prevent or treat catheter-associated infections that are frequently biofilm-related [20]. Recently, EDTA has been shown to reduce biofilm structuring and biofilm mass [16]. Similarly, colistin and other amphiphilic compounds (caspofungin and polymyxin B) were shown to reduce the exopolysaccharides content of the biofilm matrix of *Staphylococcus aureus* (Siala et al., Poster A-053 at the ICAAC 2014 in Washington, DC). Both antibiotics appear to cause matrix changes and biofilm restructuring processes leading to the loss of matrix density and visible swelling of the biofilm. Whereas the primarily bacteriostatic nitroxoline and lower concentrations of the bactericidal colistin increased the thickness of the viable biofilm, higher concentrations of the bactericidal colistin markedly decreased the thickness of the viable biofilm; the increase in thickness was primarily attributable to dead cells (Fig 7, S4 Fig).

Summarizing, the live/dead staining by SYTO9 and propidium iodide offers an excellent method for analysis of antibiotic effects on biofilms. But it has to be considered that both dyes stain the same target and unequal staining properties were observed, particularly in *P*. *aeruginosa* biofilms [21]. Compared with propidium iodide, SYTO 9 stained dead cells 18 times stronger, but it also showed strong bleaching effects. The accurate determination of the viable cells (*N*_*viable*_) different staining intensities seemed not disturb the because the two channels are evaluated separately based on local signal maxima and overlapping signals are estimated as dead cells and subtracted from the viable count. However, this fact might impact the binary parameters; *A*, *SD* and *Ʌ*.

#### Comparison of the performance with other methods

We compared if the effects observed by qBA for the antibiotic treated biofilms correlated with two standard methods (Fig 8 and S1 Table): the determination of viable cells on agar plates (CFU/mL-method) and crystal violet-based colorimetric method. Thereby, *N*/cm^2^ (Fig 8A and 8B) corresponds to CFU/mL (Fig 8B and 8C) and *A* (Fig 8E and 8F) corresponds to the absorption (Fig 8G and 8H). It has to be mentioned, that dead cells within the biofilm are only reflected by the qBA parameters *N*_*dead*_ and *A*_*red*_. Dead cells cannot be determined by the CFU/mL-method, and it must be assumed that the absorption measured by the crystal violet method is also a poor predictor of the number of dead cells. Therefore, it was inadequate to compare these parameters with the standard methods.

The observed changes in *N*_*viable*_/cm^2^ and *A*_*green*_ determined by qBA for the nitroxoline treatment perfectly correlated with the crystal violet absorption (*r* = 0.998 and *r* = 0.997, respectively). However, their correlations with the CFU/mL was reduced to *r* = 0.844 and *r* = 0.86, respectively. Nearly no correlation was found between the crystal violet absorption and *A*_*green*_ (*r* = 0.220) and between the CFU/mL and *A*_*green*_ (*r* = 0.127) for colistin treated biofilms. We hypothesize that this is because of the bactericidal effect of colistin combined with the staining procedures: crystal violet stains (exo)polysaccharides and SYTO9 nucleic acids. Colistin destabilizes bacterial membranes leading to cell lysis and release of nucleic acids, resulting in enlarged green- and red-stained areas, as observed in the CLSM Z-stack (Fig 6). This effect was observed as increased values of *A*_*green*_ and *A*_*red*_. At higher colistin concentration, this effect was reduced because the majority of the cells were lysed after 3.5 h of antibiotic treatment and the signals of stained nucleic acids dispersed. The binarization and determination of respective areas (*A*) depends on the centroids (local signal maxima) as starting points that could not be assigned due to the dispersed signals. Simultaneously, this procedure allowed a more accurate correlation of *N*_*viable*,_ with the CFU/mL (*r* = 0.775) and the crystal violet absorption (*r* = 0.813).

Summarizing, all methods should yield comparable results for biofilm treatment with primarily bacteriostatic antibiotics such as nitroxoline. For bactericidal antibiotics results obtained by the different methods might differ greatly. In general, the *N*_*viable*_/cm^2^ correlated most strongly with the CFU/mL. However, the CFU/mL might be limited by the facts that biofilms frequently contain small colony variants and dormant cells which formation is enhanced by antibiotic treatment. These may not always grow in culture. In this context, *N*_*viable*_/cm^2^ and *N*_*dead*_/cm^2^ appear to be better predictors of antibiotic effectiveness.

## Conclusions and Outlook

Biofilm-associated infections are a major problem in routine diagnostics and clinical practice. Because biofilm formation can increase antibiotic resistance up to 1,000-fold, antibiotic treatment selected according to routine MIC testing frequently results in treatment failure. Despite a study by Hall-Stoodley (2012) that presented some useful criteria for a biofilm-oriented routine diagnostic [22], currently no consensus on diagnostic guidelines for biofilm-associated infections exists. Primary hurdles are non-automated and time-consuming methods that often yield imprecise and non-reproducible results.

In the present work, we introduced a novel computerized algorithm for biofilm analysis that allows direct quantification of viable and dead bacterial cells in biofilms. The latter can currently not be measured by other methods that are based on quantification of matrix content or the counting of viable cells on agar plates. The observed biofilm changes caused by antibiotic treatment clearly indicate that indirect biofilm quantifying methods may be difficult and prone to failures because of antibiotic effects on the cells and matrix coherence.

The qBA algorithm yields results that are comparable to the microbiological CFU/mL determination but allows a faster and direct analysis with a higher throughput. The qBA protocol additionally offers various parameters that can be consulted for refining the analysis. The primary advantage of this algorithm is its ability to distinguish viable from dead cells and to generate histograms of viable and dead cells, visualizing their biofilm distribution. Despite the current Z-allocation of the cells was unsatisfactory and has to be improved, the obtained cell counts of biofilms with moderate densities yielded results comparable with other microbiological methods. The 3D analysis remains generally problematic in highly dense biofilms and in those with clumping cell clusters. Here, the 3D analysis seems to be more adequate for extrapolation of the maximum biofilm thickness. The fixed parameter 2/w must be improved and changed to an adjustable parameter that is independent of the *Prolongation* considering the Z-distances of the images. Moreover, a suitable *in silico* data set has to be design to more precisely estimate the 3D performance.

Despite some obstacles that have to be overcome, this is a first try to develop an easy but diagnostically conclusive tool that will allow insights into the mechanism of action and help to quantify the biofilm effectiveness of the respective drug. We did not address “*Lacunarity*,*”* a parameter of the homogeneity of the biofilm mass. In our opinion, this parameter is more appropriate for comparing different biofilm structures than for accessing clinically relevant parameters such as MBIC and MBEC. However, this parameter is implemented in qBA and accessible for advanced biofilm analysis.

Selecting antibiotic(s) with the highest activity against a respective biofilm will help to improve the outcomes of patients with biofilm-associated infections. This improvement requires measuring MBIC and MBEC in addition to the “planktonic” MIC. This algorithm may contribute to the development of a respective automated work flow, delivering reliable and accurate results. However, studies correlating MBIC and MBEC to treatment failure are sparse, and the methodical limitations of the current methods may contribute to this gap in knowledge. Currently, we are planning a clinical study to investigate the correlation between MBIC and treatment failure using the presented algorithm.

The latest version of the qBA algorithm and the bacteria generator can be downloaded at http://software.infectognostics-jena.de. The corresponding source code can be provided upon request from the corresponding author. Currently, the algorithm performs well on biofilms with more homogenous cell distribution, but we work on some refinements, such as improved identification and allocation of cells spanning throughout neighboring layers by replacing the constant *w*/2 by a variable and self-adjusting parameter. A more user-friendly interface and automation to decrease investigator dependency and to increase accuracy and reproducibility is also in progress.

## Supporting Information

S1 Fig Examples for the simulated data sets and the counting accuracy. A) Rods of 10 x 30 pixels in size, declension minimum and maximum = 1; B) Cocci of 10 pixels in diameter, declension minimum and maximum = 1; C) and D) identified grayscale maxima with the Z-layers (C corresponds to A, and D corresponds to B). The estimated cell numbers (10 to 10,000) are indicated on the right site of the corresponding layers.
(TIF)

S2 Fig Effects of the declension variations on the color gradients. (A-C) Cocci of 10 pixels in diameter; (D) Rods of 10 x 30 pixels in size, declension varies: in (A) Minimum and maximum declension = 0; in (B) and (D) Minimum and maximum declension = 1; in (C), Minimum declension = 0 and maximum declension = 1. The images show zoomed sections of the simulated images.
(JPG)

S3 Fig Projection images of the images sets used for analysis of allocation accuracy by *w*/2. Coccal cells (diameter 10 pixels with highest declension) of two images were shifted by 10 pixels (A) or 5 pixels (B). (C) The cells (diameter 10 pixels with highest declension) of three images were moved by 5 and 10 pixels. The allocated cells are shown in black and the red arrows indicate the allocation of the shifted cells in the illustrations.
(TIF)

S4 Fig Mean biofilm thickness *D* of *P*. *aeruginosa* biofilms treated by PBS or various concentrations of nitroxoline and colistin. (A) Nitroxolin treatment. (B) Colistin treatment. *D* was determined as described in the Materials and Methods section from the highest green (*D*_*green*_, dark green columns) and red signals (*D*_*red*_, dark red columns) of the Z-stack. The respective standard deviation of *D* (*SDD*) is only shown in the positive direction (light green or light red bars, corresponding to red and green bars). The error bars indicate the standard deviation of three replicates.
(JPG)

S1 Table Correlation factors (*r*) of the curve progressions between the different parameters and methods. *N*_*viable*_ and *A*_*green*_ represent parameters of qBA and CFU/mL was determined on agar plates and *Abs* represents absorption of crystal violet staining. The correlation factors were determined from averaged values. Less useful correlations were not provided (crossed cells); CFU = colony forming units.
(PDF)
