# Supplementary figures and images for: A Novel Computerized Cell Count Algorithm for Biofilm Analysis
Source: PLoS One. 2016 May 5;11(5):e0154937. doi: 10.1371/journal.pone.0154937 (PMC4858220; doi:10.1371/journal.pone.0154937)

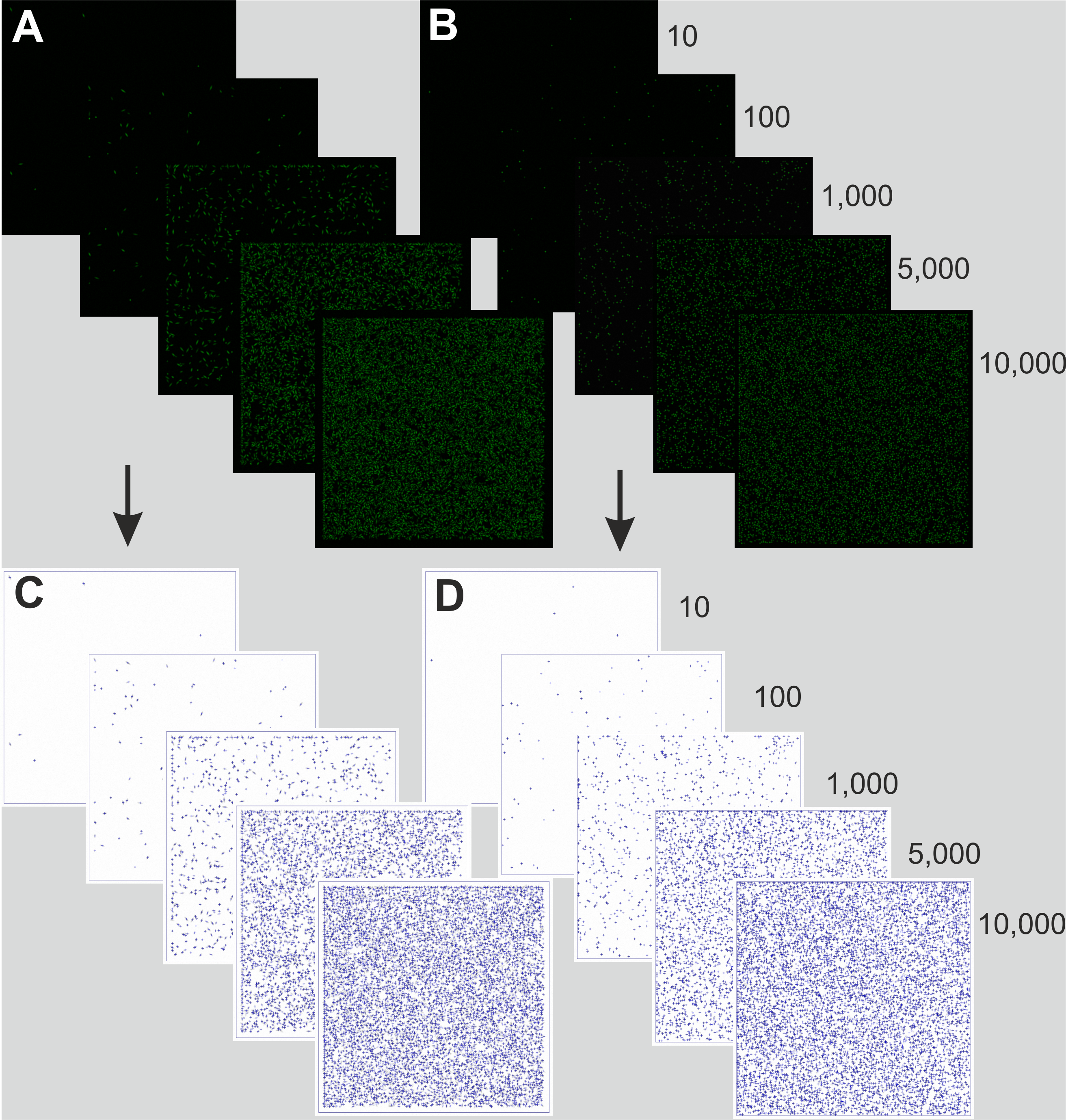

Supplement: S1 Fig — A) Rods of 10 x 30 pixels in size, declension minimum and maximum = 1; B) Cocci of 10 pixels in diameter, declension minimum and maximum = 1; C) and D) identified grayscale maxima with the Z-layers (C corresponds to A, and D corresponds to B). The estimated cell numbers (10 to 10,000) are indicated on the right site of the corresponding layers. (TIF) [file pone.0154937.s001.tif]

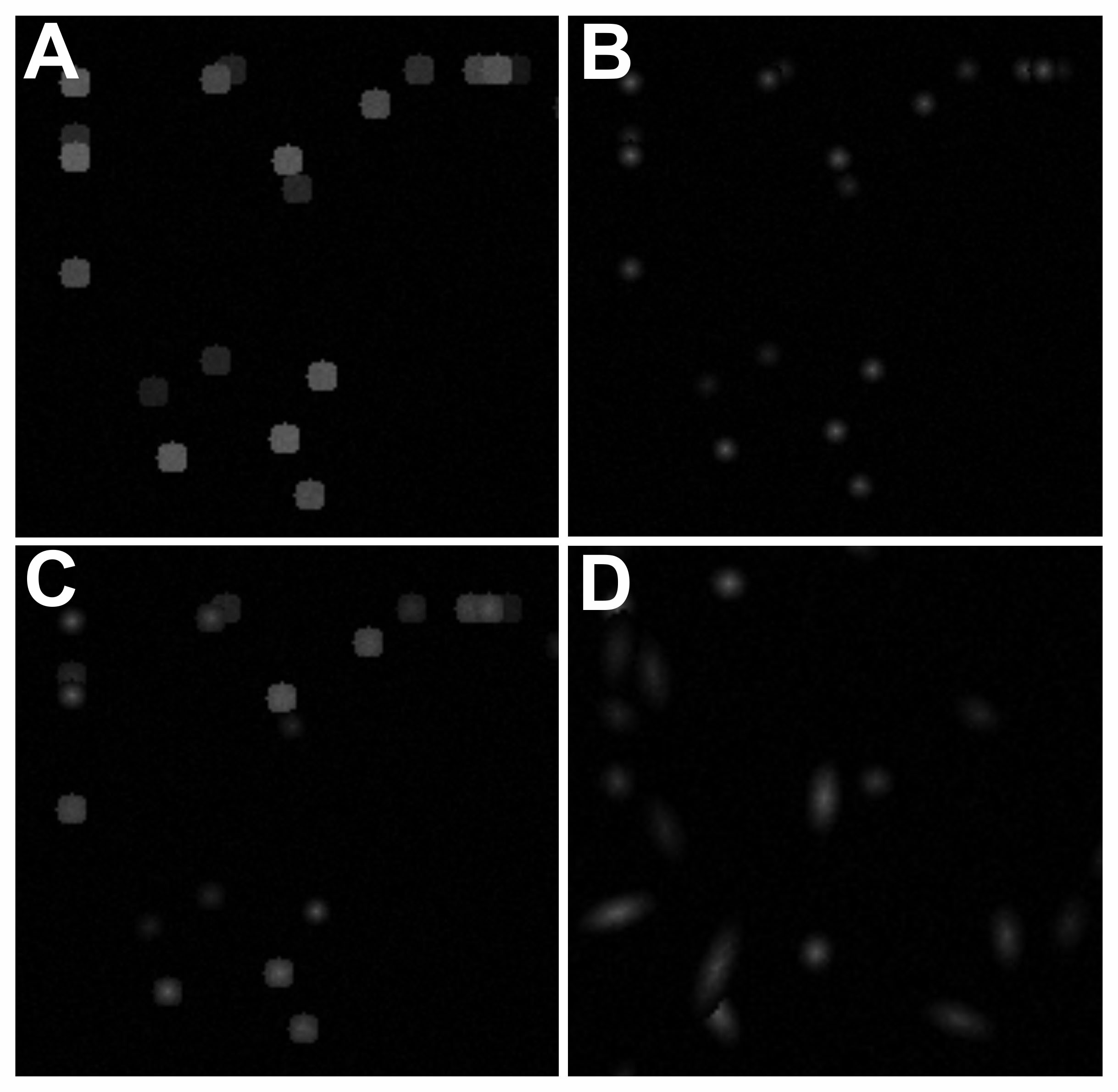

Supplement: S2 Fig — (A-C) Cocci of 10 pixels in diameter; (D) Rods of 10 x 30 pixels in size, declension varies: in (A) Minimum and maximum declension = 0; in (B) and (D) Minimum and maximum declension = 1; in (C), Minimum declension = 0 and maximum declension = 1. The images show zoomed sections of the simulated images. (JPG) [file pone.0154937.s002.jpg]

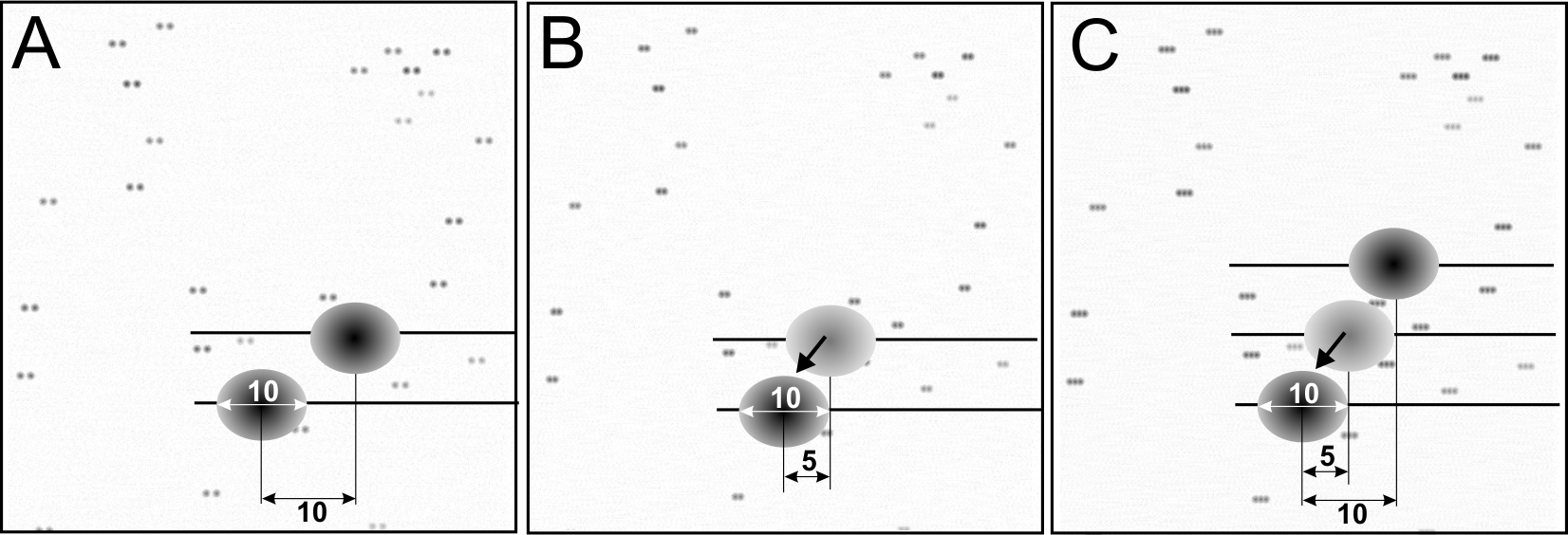

Supplement: S3 Fig — Coccal cells (diameter 10 pixels with highest declension) of two images were shifted by 10 pixels (A) or 5 pixels (B). (C) The cells (diameter 10 pixels with highest declension) of three images were moved by 5 and 10 pixels. The allocated cells are shown in black and the red arrows indicate the allocation of the shifted cells in the illustrations. (TIF) [file pone.0154937.s003.tif]

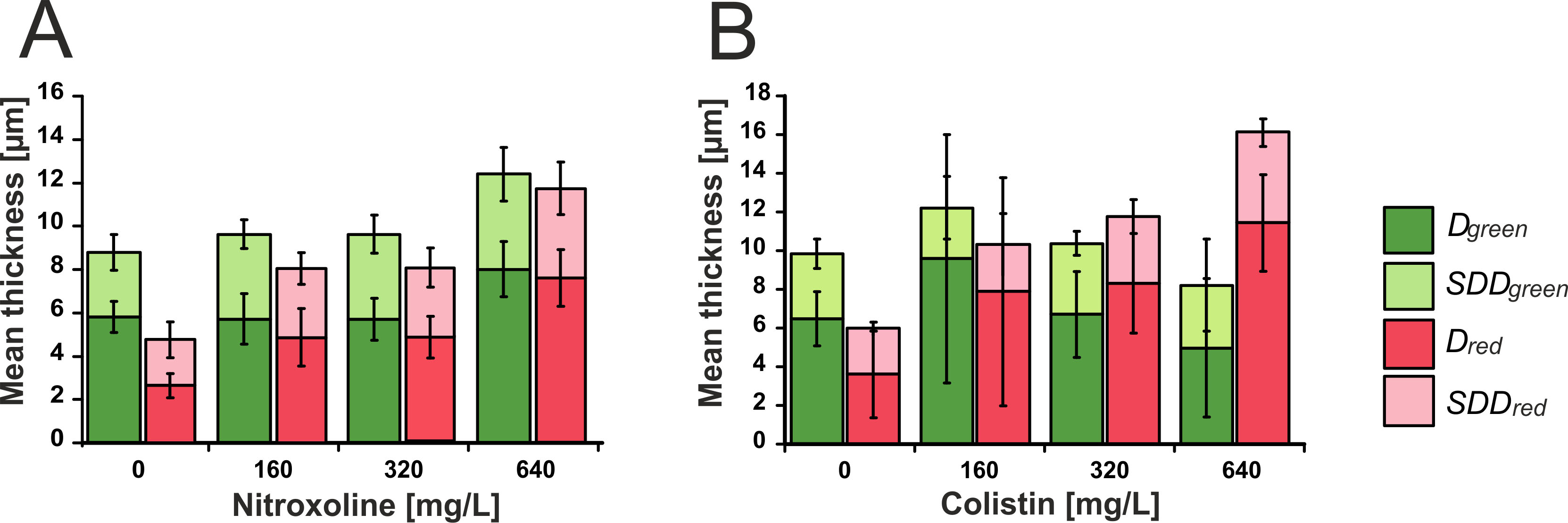

Supplement: S4 Fig — (A) Nitroxolin treatment. (B) Colistin treatment. D was determined as described in the Materials and Methods section from the highest green (Dgreen, dark green columns) and red signals (Dred, dark red columns) of the Z-stack. The respective standard deviation of D (SDD) is only shown in the positive direction (light green or light red bars, corresponding to red and green bars). The error bars indicate the standard deviation of three replicates. (JPG) [file pone.0154937.s004.jpg]
