# Supplementary material for: A Novel Computerized Cell Count Algorithm for Biofilm Analysis
Source: PLoS One. 2016 May 5;11(5):e0154937. doi: 10.1371/journal.pone.0154937 (PMC4858220; doi:10.1371/journal.pone.0154937)
Supplement: S1 Table — Nviable and Agreen represent parameters of qBA and CFU/mL was determined on agar plates and Abs represents absorption of crystal violet staining. The correlation factors were determined from averaged values. Less useful correlations were not provided (crossed cells); CFU = colony forming units. (PDF) [file pone.0154937.s005.pdf]

S1 Table. Correlation factors (*r*) of the curve progressions between the different parameters and methods.

	N_{viable}	A_{green}	N_{dead}	A_{red}	*Abs*
Colistin					
A_{green}	0.719				
N_{dead}	-0.321	/			
A_{red}	/	0.391	0.999		
Abs	0.813	0.220			
CFU/mL	0.775	0.127			0.988
Nitroxoline					
A_{green}	0.998				
N_{dead}	-0.703	/			
A_{red}	/	-0.863	0.978		
Abs	0.998	0.997			
CFU/mL	0.844	0.866			0.828

N_{viable} and A_{green} represent parameters of qBA and CFU/mL was determined on agar plates and *Abs* represents absorption of crystal violet staining. The correlation factors were determined from averaged values. Less useful correlations were not provided (crossed cells); CFU = colony forming units.
